# Evaluation of the Photocatalytic Efficiency of Silver Nanoparticles Synthesized by Green Chemistry in the Degradation of Methyl Orange

**DOI:** 10.3390/molecules31203672

**Published:** 2026-10-14

**Authors:** José Antonio Barraza Madrigal, Jesús Israel Guzmán Castañeda, Diana Hernández Bautista, Karla Jenny Lozano Rojas, Manuela Díaz Cruz, Elsa Rubio Espino

**Affiliations:** 1 Academia de Física, Instituto Politécnico Nacional-ESIQIE, Edif. 6, Mexico City 07738, Mexico; dhernandezb1906@alumno.ipn.mx; 2 Academia de Química Analítica, Instituto Politécnico Nacional-ESIQIE, Edif. Z6, Mexico City 07738, Mexico; klozanor@ipn.mx; 3 Departamento de Ingeniería en Metalurgia, Instituto Politécnico Nacional-ESIQIE, Mexico City 07738, Mexico; mdiazc@ipn.mx; 4 Laboratorio de Robótica y Mecatrónica, Instituto Politécnico Nacional-CIC-IPN, Mexico City 07738, Mexico; erubio@cic.ipn.mx

**Keywords:** green synthesis, silver nanoparticles, photocatalytic degradation, methyl orange, gamma-alumina

## Abstract

The release of methyl orange (MO) into bodies of water represents a serious environmental problem due to its chemical stability, toxicity, and persistence. To address this issue, this work presents the green synthesis of silver nanoparticles (AgNPs) using natural extracts of rose leaves (RL), rose petals of the pink Castile rose (PCR), and common yellow rose petals (YRP) as reducing agents. The formation of the AgNPs was confirmed by ultraviolet–visible (UV-Vis) spectroscopy through the localized surface plasmon resonance (LSPR) band and Transmission Electron Microscopy (TEM) obtaining sizes smaller than 15 nm, while FTIR analysis confirmed the presence of phenols, flavonoids, and proteins, which are suggested by spectral shifts to act as reducing and stabilizing agents. After optimizing the synthesis, the AgNPs were supported on gamma-alumina (γ-Al_2_O_3_). Gamma-alumina is characterized by a large surface area and high chemical and thermal stability; it inhibits nanoparticle agglomeration, thereby significantly improving electron-hole pair separation in photocatalytic processes, ultimately forming AgNPs/γ-Al_2_O_3_ photocatalysts. The activity of these composites was evaluated in the degradation of MO under UV irradiation (254 nm) and white light LED (380–750 nm). The catalysts demonstrated a dye removal trend, achieving degradation efficiencies under ultraviolet light of 4.94% (RL), 24.69% (YRP), and 60.49% (PCR); these figures increased under white LED light to 43.21% (RL), 64.20% (YRP), and 76.54% (PCR) after 60 min. It is hypothesized that this enhanced performance under visible light arises from mechanisms driven by reactive oxygen species (ROS) boosted by the LSPR effect, as has been documented in similar plasmonic systems. It is concluded that these supported photocatalysts constitute an environmentally friendly, sustainable, and alternative for wastewater treatment.

## 1. Introduction

Water pollution from persistent organic compounds, such as synthetic dyes commonly used in textile industries, is one of the most relevant problems worldwide. These dyes have highly stable structures, low biodegradability, and high water solubility, which makes their elimination difficult by means of conventional wastewater treatment methods [1].

Among these compounds, methyl orange (MO) stands out as a widely used dye, which is usually used as a model of pollution, as it is highly resistant to degradation and potentially toxic to aquatic organisms and human health [2].

Oxidation processes, such as photocatalysis, have emerged as efficient and sustainable alternatives for degradation and mineralization of organic pollutants under light irradiation [3]. This process is based on the use of photoreactive materials capable of absorbing energy and generating reactive oxygen species (ROS), such as hydroxyl and superoxide radicals, which promote the progressive oxidation of pollutants until less toxic products are obtained, such as carbon dioxide (CO_2_) and water (H_2_O). In this context, silver nanoparticles (AgNPs) have attracted considerable interest due to their optical properties derived from the localized surface plasmon resonance (LSPR) band, as well as their high catalytic activity and their ability to enhance the photoactive response of various support materials [4,5].

Previous research has demonstrated the potential of supporting AgNPs on gamma-alumina (*γ*-*Al*_2_*O*_3_) to improve catalytic performance [6,7]. Nonetheless, commonly reported methods rely on chemical reduction requiring hazardous reagents, complex equipment’s, and high energy consumption, which limits their environmental sustainability [8,9]. Green synthesis methods have been widely documented for unsupported AgNPs; the synthetization of AgNPs onto *γ*-*Al*_2_*O*_3_ matrices, specifically for photocatalytic degradation, remains scarcely explored. In that sense, developing an environmentally friendly approach to fabricate these supported composites, to overcome toxicity and cost limitations of the conventional methods, will be desirable.

In this work, aqueous extracts of rose leaves (RL), rose petals of the pink Castile rose (PCR) and common yellow rose petals (YRP) were used as a rich source of secondary metabolites, such as phenols and flavonoids. The specific functional groups of these metabolites act as reducing and stabilizing agents for the green synthesis of AgNPs, aiming for a sustainable approach [10,11]. Subsequently, the synthesized AgNPs were supported on gamma-alumina (γ-Al_2_O_3_), a material known to improve nanoparticle dispersion, stability, and recoverability, while also promoting photocatalytic performance [12]. Consequently, the specific functional groups analyzed by spectroscopic techniques (such as -OH and C=O) belong to the chemical structures of these secondary metabolites; therefore, the characterization of the functional profile provides an indirect representation of the metabolic footprint driving their synthesis.

The aim of this investigation was to correlate the general phytochemical profile of diverse plant extracts with the green synthesis and the optical and structural properties of AgNPs, as well as to determine the photocatalytic efficiency of AgNPs/γ-Al_2_O_3_ materials in the photocatalytic degradation of MO under UV irradiation.

## 2. Materials and Methods

### 2.1. Regents and Instrumentation

Silver nitrate (AgNO_3_, 0.019 M) was used as the metallic precursor, gamma-alumina (γ-Al_2_O_3_) powder (average particle size 0.05 µm) as the catalytic support, and methyl orange (MO) (Acid Orange 52), Merck brand, CAS number: 547-58-0 (Merck KGaA, Darmstadt, Germany), at an initial concentration of 5 × 10^−5^ M, as the model pollutant. An unirradiated MO solution without AgNPs was used as the control.

For the green synthesis, dried plant material was used as a reducing agent, consisting of rose leaves (RL), rose petals of the pink Castile rose (PCR), and common yellow rose petals (YRP). Heating and stirring were performed on a Corning PC4200 hot plate (Corning, NY, USA), and weighing was carried out on a Sartorius ED224S analytical balance (Göttingen, Germany).

UV-Vis spectroscopy was performed using a Perkin Elmer LAMBDA 365+ spectrophotometer (Shelton, CT, USA) equipped with quartz cuvettes. The spectra were recorded at a scan rate of $480\frac{\mathrm{nm}}{\min}$ over the wavelength range of 200–600 nm.

To characterize the AgNPs colloidal solutions, a JEOL JEM-ARM200CF Transmission electron microscope (Akishima, Japan) was used, operating at an acceleration voltage of 200 keV and equipped with a cold emission gun (CFEG).

The identification of functional groups in the dried extracts was carried out by Fourier transform infrared (FTIR) spectroscopy using a Perkin Elmer Spectrum 65 FTIR spectrometer (Shelton, CT, USA); tests were performed in the mid-infrared region (4000–800 cm^−1^).

The suspensions for the catalytic tests were introduced into a mirror-based photocatalytic reactor, operated at 110–120 V AC, equipped with a 20 W, 60 Hz UV lamp with emission at 254 nm. Furthermore, for photocatalytic evaluations under white light, a 30 W LED floodlight with emission interval at 380–750 nm and an output of 2700 lumens was used within the same reactor. The system was operated with constant airflow.

### 2.2. Preparation of Extracts

To prepare the infusions of each system, 5 g of each dried plant material (RL, PCR, and YRP) were weighed and added to 120 mL of distilled water in 250 mL beakers. The mixtures were heated to boiling (approximately 100 °C), at which point the water turned yellow or orange, indicating the release of secondary metabolites, such as phenols and flavonoids, and natural pigments, which are essential for driving the subsequent nanoparticle generation. The infusions were cooled by partial immersion in cold water, filtered to remove solid residues, and stored in amber bottles to prevent photoinduced degradation. The extracts obtained were designated as stock extracts. Subsequently, the stock extracts were diluted at a ratio of 1:100 to avoid saturation and interference in the spectrophotometric readings.

### 2.3. Green Synthesis of AgNPs and Optimization

To determine the optimal conditions for AgNPs synthesis, fifteen different volumetric combinations of plant extract and AgNO_3_ solution were evaluated for each of the three plant materials (Table 1). The volumetric ratios ranged from 4:2 to 2:4 (extract:AgNO_3_).

All formulations were synthesized independently in triplicate and monitored using UV-Vis spectroscopy. The spectra obtained from the three experimental replicates were used to evaluate nanoparticle formation based on the intensity, definition, and stability of the localized surface plasmon resonance (LSPR) band.

Following the screening of the different extract:AgNO_3_ ratios, the selected formulation was prepared using 4 mL of the plant extract and 2 mL of the AgNO_3_ solution (0.01941 N). Both solutions were heated to 100 °C, after which the AgNO_3_ solution was added to the plant extract under constant magnetic stirring at 260 rpm for 10 min. During the reaction, the color of the solution changed from yellow to brown, indicating the reduction of silver ions (*Ag*^+^) and the formation of AgNPs. This phenomenon is associated with the appearance of the LSPR band and constitutes visual evidence of nanoparticle formation through green synthesis [13,14,15]. The resulting colloidal suspensions were stored in amber tubes at room temperature until further characterization.

### 2.4. Preparation of Supported Photocatalysts (AgNPs/γ-Al_2_O_3_)

To prepare the supported photocatalysts, 0.1 g of *γ*-*Al*_2_*O*_3_ was weighed and combined with 1 mL of each optimized colloidal solution of AgNPs obtained from RL, PCR, and YRP extracts. The mixtures were carefully homogenized to promote the uniform deposition of the nanoparticles onto the *γ*-*Al*_2_*O*_3_ surface and were subsequently dried at room temperature until complete solvent evaporation. The resulting photocatalysts were designated as *AgNPs*/*γ*-*Al*_2_*O*_3_-*RL*, *AgNPs*/*γ*-*Al*_2_*O*_3_-*PCR,* and *AgNPs*/*γ*-*Al*_2_*O*_3_-YRP. It is noteworthy that for the control sample, only gamma-alumina (γ-Al_2_O_3_) was used.

### 2.5. Selection of the Working MO Solution

Initial spectrophotometric analysis revealed that the concentrated MO stock solution (5 × 10^−5^ M) shows absorption band saturation above *A* ≈ 3.0, leading to band flattening and deviation from the Beer–Lambert law. In that sense, this concentrated solution was not used for degradation tests. Instead, a systematic dilution study was evaluated to ensure that the operational absorbance remained within the linear dynamic range of the spectrophotometer (Table 2). Among the tested ratios, the 2.5:10 mL (*MO*:*distilled water*) was selected, corresponding to 2.5 mL of the MO stock solution adjusted to a final volume of 10 mL with distilled water. The final concentration of the working solution was calculated using the dilution equation presented in Equation (1).

(*C*_1_)∙(*V*_1_) = (*C*_2_)∙(*V*_2_) (1)

where “*C*_1_” is the initial concentration of the MO stock solution (5 × 10^−5^ M), “*V*_1_” is the volume of the stock solution used (2.5 mL), “*C*_2_” is the final concentration of the working solution, and “*V*_2_” is the final volume after dilution (10 mL).

Solving for “*C*_2_” and substituting the corresponding values, a final concentration of 1.25 × 10^−5^ M was obtained.

This solution was used as the control (unirradiated MO solution without AgNPs) for photocatalytic experiments, as it provided an absorption band within an appropriate range for monitoring and comparison by *UV*-*Vis* spectroscopy.

Table 2 shows the different dilutions prepared from the MO stock solution. The experimental concentration of each dilution was calculated using Equation (1). The MOD-2.5/10 dilution, with an experimental concentration of 1.25 × 10^−5^ M, was selected as the working solution for the photocatalytic experiments.

### 2.6. Photocatalytic Degradation Tests

Catalytic tests were performed by mixing 0.1 g of each supported photocatalyst with 20 mL of the MO working solution in 50 mL quartz flasks. For the control sample (MO-stock), only *γ*-*Al*_2_*O*_3_ was used, without the addition of nanoparticles and without UV radiation. The mirror-based photocatalytic reactor (Figure 1) was kept under continuous stirring for 1 h to ensure the homogeneity of the suspension. Following the degradation process, the samples were filtered using an Acrodisc with a 0.45 μm hydrophilic PTFE membrane. The mixtures were analyzed using UV-Vis spectroscopy before and after the photocatalytic process to evaluate the degradation efficiency.

The degradation efficiency was calculated from the percentage reduction in dye concentration, considering the relationship between the initial concentration “*C*_0_” and the final concentration “*C_f_*”, as shown in Equation (2). The initial baseline absorbance of the reaction mixture before irradiation was adjusted to *A* = 0.81 (<1.0), to ensure that the subsequent spectrophotometric degradation monitoring remained within the ideal linear absorbance regime of the Beer–Lambert law.

The percentage of dye degradation was calculated using Equation (2):

(2) $${\%}_{\mathrm{Degradation}}=\frac{\left(A_{0})\;-(A_{f}\right)}{{(A}_{0})}\times 100=\frac{\left(C_{0})\;-(C_{f}\right)}{{(C}_{0})}\times 100\;$$

where “*C*_0_” is the initial concentration of the dye, and “*C_f_*” is the concentration of the dye after the photocatalytic treatment.

## 3. Results and Discussion

### 3.1. Synthesis and Optimization of Silver Nanoparticles

The chromatic change observed in the solutions obtained from extracts of RL, PCR, and YRP, from yellow to brown tones, evidenced the formation of silver nanoparticles (AgNPs) and the reduction of silver ions (*Ag*^+^) to metallic silver (*Ag*^0^). This behavior, together with the appearance of the LSPR band, is characteristic of AgNPs obtained through green synthesis [13,14,16,17].

The optimal synthesis conditions were determined by evaluating fifteen volumetric variations between the plant extract and the metallic precursor AgNO_3_. Through colorimetric and spectrophotometric analysis, it was determined that the 4:2 ratio (extract: AgNO_3_) presented the highest absorbance values within each system, suggesting higher AgNPs formation efficiency under this condition and efficient use of the metallic precursor.

### 3.2. Optical and Structural Characterization

Optical characterization of the 1:100 diluted plant extracts and AgNPs was performed by UV-Vis spectroscopy. The UV-Vis spectra of the 1:100 diluted RL, PCR, and YRP extracts are presented in Figure 2. These spectra were used as a reference for the optical characterization of the plant extracts prior to the synthesis of AgNPs.

The UV-Vis spectra in Figure 2 show the absorption bands of the organic reducing agents, which exhibit prominent bands characteristic of polyphenolic structures, especially flavonoids and phenolic acids. The distinctive electronic transitions observed at 265 nm and 277 nm correspond strictly to Band II of the flavonoids, which arises form *π* → π^*^ electronic transitions [18,19]. Likewise, the shoulder extending across the 300–350 nm range represents Band I, associated with the cinnamoyl system of flavonoid aglycones and cinnamic acid derivatives [18].

The LSPR band of the AgNPs was evaluated within the 300–600 nm range, allowing clear visualization of the plasmonic peak and assessment of nanoparticle formation and colloidal stability. This spectral range is consistent with values reported in the literature [14,15,20]. Figure 3 presents the UV-Vis spectrum obtained for each of the fifteen AgNPs formulations listed in Table 1. Each formulation was analyzed in triplicate, and the spectra shown correspond to the independent experimental replicates.

Comparison of the spectral responses of the fifteen formulations revealed that the 4:2 extract:AgNO_3_ volumetric ratio exhibited the most favorable optical response, characterized by a well-defined and intense LSPR band. Therefore, the 4:2 ratio was selected as the optimal synthesis condition.

Figure 4 presents the UV-Vis spectra of the AgNPs synthesized under the optimized 4:2 extract:AgNO_3_ volumetric ratio using RL, PCR, and YRP extracts. The observed LSPR bands confirm the formation of AgNPs under the selected synthesis conditions.

Transmission electron microscopy (TEM) was used to characterize the morphology and size distribution of the AgNPs, as shown in Figure 5. Bright-field micrographs confirmed the successful formation of predominantly spherical or quasi-spherical silver nanoparticles via all three green synthesis routes; these particles exhibited well-defined crystal lattices. Figure 5a (sample synthesized with RL extract) shows highly dispersed, isolated, and uniform nanoparticles with the smallest average diameters, ranging from 3 to 10 nm. In contrast, sizes range from 8 to 12 nm for the YRP sample (Figure 5b), while the PCR sample (Figure 5c) shows predominant sizes between 5 and 15 nm. A clear correlation was observed between the microstructural characteristics obtained via TEM and the *UV*-*Vis* spectra; maximum absorption peaks at shorter wavelengths corresponded to smaller particle sizes, consistent with the LSPR effect. The sizes obtained were analyzed using the ImageJ program on an estimated 200 particles.

Fourier transform infrared (FTIR) spectroscopy, for the identification of functional groups in the dried extracts, was performed in the mid-infrared region (4000–800 cm^−1^), where the fundamental vibrations associated with most organic functional groups present in the extracts are observed [21,22]. The FTIR spectra of the dried RL, PCR, and YRP extracts are presented in Figure 6.

The color change observed in all fifteen formulations (Figure 7), from yellow to brown tones, confirmed the reduction process described in Section 3.1. The synthesized AgNPs were then characterized by *UV*-*Vis* spectroscopy and FTIR analysis.

Figure 4 shows the UV-Vis spectra of AgNPs synthesized at the optimal 4:2 volumetric ratio using RL, PCR, and YRP extracts. The formation of AgNPs was confirmed by the characteristic LSPR band, with *λ_max_* values of 424 nm (RL), 429 nm (PCR), and 426 nm (YRP). These values are consistent with those reported for AgNPs obtained by green synthesis (418–450 nm) [14,15,17,20,23], confirming successful nanoparticle formation.

Among the evaluated systems, AgNPs synthesized using PCR extract exhibited the highest absorbance intensity (1.76) compared with RL (1.04) and YRP (1.39), indicating a hyperchromic effect and suggesting a higher nanoparticle concentration. As evidenced by the vertical reference markers in the full spectra (Figure 4), AgNPs synthesized using PCR extract displayed the most pronounced hyperchromic effect. Additionally, a slight bathochromic shift (red shift) was observed for PCR (429 nm) compared with RL (424 nm), while YRP showed an intermediate shift (426 nm). This shift is associated with differences in nanoparticle size and/or morphology, as well as changes in the dielectric environment around the AgNPs due to the adsorption of organic compounds from the extract [24,25].

The higher synthesis yield obtained with PCR (evidenced by the hyperchromic effect) correlates with the higher absorbance of the PCR extract in *UV*-*Vis* (0.78 at 265 nm, Figure 2), indicating a greater availability of phenolic and flavonoid compounds with reducing capacity. However, the bathochromic shift suggests that AgNPs-PCR might present a slightly larger average size or higher aggregation compared with RL and YRP, which could be influenced by differences in the type and relative abundance of phenolic compounds, including anthocyanin pigments characteristic of rose petals of the pink Castile rose [25].

Overall, these results indicate that the botanical source influences the optical properties and nanoparticle formation process of AgNPs, with PCR extract showing the most pronounced optical response under the evaluated conditions.

FTIR analysis of the dried plant samples RL, PCR, and YRP revealed the presence of functional groups associated with secondary metabolites (Figure 6). Broad and intense bands around 3400 cm^−1^ were attributed to O-H stretching from phenolic compounds, flavonoids, and tannins, while signals in the 1730–1650 cm^−1^ and 1620–1580 cm^−1^ regions corresponded to C = O, aromatic C = C, and amide vibrations associated with polyphenols and proteins. The differences in the optical and morphological properties of the synthesized AgNPs are attributable to the relative quantity of functional groups present in each plant extract. The FTIR spectra of the PCR extract exhibited the highest intensity in the stretching bands corresponding to hydroxyl (-OH) and carbonyl (C=O) groups; this suggests a higher content of polyphenols and flavonoids, which facilitates more efficient reduction during nanoparticle synthesis. This high concentration of active metabolites accelerates the reaction kinetics of *Ag*^+^ ions, resulting in a greater yield of nanoparticles—a finding that accounts for the observed hyperchromic effect (absorbance of 1.76). The YRP extract exhibited an intermediate density of these functional groups, which favored a somewhat more limited nucleation and growth process, resulting in an intermediate bathochromic shift (426 nm). Finally, although the RL extract has a lower concentration of reducing polyphenols, it is particularly rich in structural proteins and complex polysaccharides that act as stabilizers. These biomolecules prevent excessive growth during synthesis, generating smaller and more uniform particles; this is associated with the narrower and more symmetrical LSPR band observed at 424 nm. After AgNP synthesis, these bands showed slight shifts toward lower wavenumbers and decreased intensity, suggesting the involvement of these metabolites as reducing and stabilizing agents on the AgNP surface [26,27]. The *UV*-*Vis* analysis of the extracts showed that PCR presented the highest absorbance at 265 nm (0.78) compared with YRP (0.71) and RL (0.35), which is consistent with the higher AgNPs formation efficiency observed for this extract. Although the spectral changes suggest the involvement of these metabolites in the reduction process, further experiments such as analytical isolation would be required to identify the specific molecules responsible.

### 3.3. Photocatalytic Activity in the Degradation of Methyl Orange

The UV-Vis spectra obtained from the prepared MO dilutions, as well as the comparison between the stock solution and the selected working solution, are shown in Figure 8a,b. The calibration curve constructed from the absorbance values of the diluted MO solutions is presented in Figure 8c.

The dye concentration was determined from the calibration curve obtained by UV-Vis spectroscopy, according to the linear equation presented in Equation (3).

*A* = (126,161)∙(*C*) − (0.0763) (3)

where

“*A*” is the absorbance, “*C*” is the concentration of the dye (*M*); the coefficient of determination obtained was (R^2^ = 0.9892), indicating adequate linearity and reliability of the model.

Solving for the concentration, we obtain Equation (4):

(4) $$C\;=\frac{\left(A)\;+(0.0763\right)}{\left(126,161\right)}\;$$

Equation (3) was used to determine the linear working range for the initial concentrations of the MO stock dye and to ensure compliance with the Beer–Lambert law. It should be noted that the calibration curve and blank photolysis measurements were performed at a working wavelength of 505 nm, with the dye in its protonated (acidic) form, prior to contact with the gamma-alumina.

To determine the chemical stability of OM under UV radiation and white light, a photolysis test was conducted. Light irradiation was performed in the absence of catalysts to establish a baseline and subsequently compare the observed photocatalytic activity with that obtained when using catalysts. This control procedure allows us to visualize how catalysts contribute to the induced degradation process versus degradation caused exclusively by photons.

Figure 9 illustrates the UV-Vis spectroscopy results from the control experiments (conducted without a photocatalyst), which show an absorption maximum at 505 nm, a characteristic feature of OM. It is observed that, after 60 min of exposure to white light, the dye undergoes virtually no degradation; this confirms the high chemical stability of the OM structure when exposed to low-energy photons within the visible spectrum. In contrast, a slight decrease in concentration was observed after the same period under ultraviolet (UV) light. This minor photolytic decomposition is attributed to the fact that UV radiation has a shorter wavelength and, consequently, higher energy per photon; while this can cause the breaking or degradation of certain weak molecular bonds in the OM, the overall degree of degradation remains almost imperceptible.

Figure 10 shows the UV-Vis spectra of the MO working solution before the photocatalytic treatment (0 min) and after 60 min of UV irradiation in the presence of the *AgNPs*/*γ*-*Al*_2_*O*_3_-*RL*, *AgNPs*/*γ*-*Al*_2_*O*_3_-*PCR*, and *AgNPs*/*γ*-*Al*_2_*O*_3_-*YRP* composites. A decrease in the characteristic absorption band of MO at *λ_max_* = 463 nm was observed after the photocatalytic process, indicating a reduction in the dye concentration in the aqueous medium. Methyl orange is an azo pH indicator; its protonated (acidic) form exhibits maximum absorbance at 505 nm, but upon contact with gamma-alumina, it shifts to its deprotonated form, resulting in a hypsochromic shift (“blue shift”) to 463 nm. Gamma-alumina typically has a pH between 7.5 and 9.0; when it comes into contact with an acidic medium (such as methyl orange), it acts as a free-proton acceptor, forcing the methyl orange to deprotonate and become more basic, thereby causing the shift. Furthermore, the decrease in the initial baseline absorbance from A = 1.43, recorded for the pure working solution at 505 nm, to A = 0.81 at 463 nm (*t* = 0 min) before irradiation is attributed to two effects: the lower molar absorptivity (*ϵ*) of the deprotonated anionic azo species compared to its protonated form, and the rapid dark adsorption of dye molecules onto the high-surface-area *γ*-*Al*_2_*O*_3_ support upon suspension. Thus, this absorbance measured at *t* = 0 min (*A* = 0.81) was established as the baseline (*C_o_*) after reaching adsorption-deprotonation equilibrium.

The experimental absorbance values obtained from UV-Vis spectra after 60 min of UV treatment were 0.77 for AgNPs/γ-Al_2_O_3_-RL, 0.61 for AgNPs/γ-Al_2_O_3_-YRP, and 0.32 for AgNPs/γ-Al_2_O_3_-PCR, starting from an initial reference absorbance of 0.81 recorded at t = 0 min. The photocatalytic degradation efficiency of each composite system was calculated using Equation (2), utilizing these operational absorbance values at 463 nm. Since Equation (2) mathematically normalizes the measurement via the absorbance ratio, it is possible to accurately determine the degradation percentage using only raw absorbance data, without the need for absolute concentration data derived from a calibration curve. This approach is consistent with standard treatments for pseudo-first-order kinetic data under a continuous deprotonation equilibrium.

The calculated photocatalytic degradation efficiencies after 60 min of irradiation were 4.94% for *AgNPs*/*γ*-*Al*_2_*O*_3_-*RL*, 60.49% for *AgNPs*/*γ*-*Al*_2_*O*_3_-*PCR*, and 24.69% for *AgNPs*/*γ*-*Al*_2_*O*_3_-*YRP*. Among the evaluated materials, *AgNPs*/*γ*-*Al*_2_*O*_3_-*PCR* exhibited the highest photocatalytic activity, showing the greatest reduction in MO concentration under the experimental conditions. In contrast, *AgNPs*/*γ*-*Al*_2_*O*_3_-*RL* showed limited photocatalytic degradation efficiency, suggesting a lower interaction between the synthesized AgNPs and the reaction system.

Figure 11 presents the photocatalytic degradation tests of MO under white LED light irradiation for 60 min, using the same three catalysts. The UV-Vis absorption spectra show a significant improvement in dye removal compared to the photolysis experiments illustrated in Figure 9. The results obtained using white light showed that the maximum absorbance at 463 nm decreased from an initial value of 0.81 to 0.46, 0.29, and 0.19 for AgNPs/γ-Al_2_O_3_-RL, AgNPs/γ-Al_2_O_3_-YRP, and AgNPs/γ-Al_2_O_3_-PCR, respectively. These values correspond to calculated photocatalytic degradation efficiencies of 43.21% for the composite obtained with RL, 64.20% for the YRP system, and a remarkable 76.54% for the photocatalyst synthesized with PCR.

The results obtained for the two irradiation systems (UV light and white light) follow the same trend regarding the degradation order of the catalysts used (PCR > YRP > RL), albeit with a substantial increase in efficiency under white light. For example, the AgNPs/γ-Al_2_O_3_-PCR sample showed an increase in removal capacity from 60.49% under UV light to 76.54% under white light. This performance improvement under visible light irradiation is largely attributed to the localized surface plasmon resonance (LSPR) effect; this phenomenon induces the resonant excitation of conduction electrons on the surface of the nanoparticles. The broad LSPR bands, centered between 424 and 429 nm (Figure 4), overlap significantly with the emission spectrum of the white LED light.

The experimental absorbance values and degradation percentages for the OM from both irradiation systems are presented in Table 3.

Differences in photocatalytic performance, in addition to the type of light used, can be attributed to the properties of each extract, which influence the formation, stabilization, and surface properties of AgNPs. The photocatalytic performance of *AgNPs*/*γ*-*Al*_2_*O*_3_-*PCR* (60.49%) correlates with the higher absorbance of the PCR extract in *UV*-*Vis* (0.78 at 265 nm) and with the higher AgNPs synthesis yield (hyperchromic effect, 1.76), suggesting that the components present in this extract favor the formation of nanoparticles with higher surface area and better interaction with the *γ*-*Al*_2_*O*_3_ support [24] a trend observed regardless of the light source used. Additionally, the bathochromic shift observed for AgNPs-PCR (429 nm) indicates differences in the surface environment of the nanoparticles, possibly due to the adsorption of specific organic compounds that could enhance charge transfer and the generation of reactive oxygen species (ROS) involved in dye degradation [25].

These factors affect nanoparticle dispersion on the *γ*-*Al*_2_*O*_3_ support and the availability of active sites [28,29,30]. Moreover, AgNPs promote photoinduced charge transfer and ROS, enhancing MO degradation through oxidation of its azo chromophore.

## 4. Conclusions

Silver nanoparticles (AgNPs) were successfully synthesized using aqueous extracts obtained from rose leaves (RL), rose petals of the pink Castile rose (PCR), and common yellow rose petals (YRP) through a green and sustainable approach. The formation of AgNPs was confirmed by the characteristic localized surface plasmon resonance (LSPR) bands at 424 nm (RL), 429 nm (PCR), and 426 nm (YRP). FTIR analysis indicated the presence of functional groups associated with phenolic compounds, flavonoids, and proteins, whose spectral shifts suggest their involvement in the reduction of silver ions (Ag+) and the stabilization of the nanoparticles.

The *AgNPs*/*γ*-*Al*_2_*O*_3_ composites exhibited photocatalytic activity toward methyl orange (MO) degradation under *UV* and white light irradiation. Among the *AgNPs*/*γ*-*Al*_2_*O*_3_ photocatalysts, *AgNPs*/*γ*-*Al*_2_*O*_3_-*PCR* showed the highest photocatalytic performance, achieving 60.49% degradation after 60 min, whereas *AgNPs*/*γ*-*Al*_2_*O*_3_-*YRP* and *AgNPs*/*γ*-*Al*_2_*O*_3_-*RL* reached degradation efficiencies of 24.69% and 4.94%, respectively. While 76.54% degradation was achieved with white LED illumination using AgNPs/γ-Al_2_O_3_-PCR over the same period, the AgNPs/γ-Al_2_O_3_-YRP and AgNPs/γ-Al_2_O_3_-RL systems also showed improvement, degrading 64.20% and 43.21%, respectively.

Among the prepared catalysts, the AgNPs/γ-Al_2_O_3_-PCR system consistently demonstrated superior photocatalytic performance under both irradiation systems, achieving 60.49% degradation with UV light and a higher efficiency of 76.54% with white LED illumination after 60 min. Under visible light, the AgNPs/γ-Al_2_O_3_-YRP and AgNPs/γ-Al_2_O_3_-RL systems also exhibited higher efficiency compared to UV light. Furthermore, these results confirm that the plasmonic properties of the AgNPs synthesized in this study significantly enhance degradation capacity compared to photolysis alone.

These results indicate that the properties of the extracts obtained from different plant materials play an important role in the formation and photocatalytic performance of the synthesized AgNPs.

Enhanced activity of $\frac{\mathrm{AgNPs}}{\gamma }\text{-}Al_{2}O_{3}\text{-}\mathrm{PCR}$ can be attributed to the synergistic interaction between the AgNPs and the *γ*-*Al*_2_*O*_3_ support, which favors nanoparticle dispersion and, according to the literature, promotes the generation of reactive oxygen species (ROS) involved in degradation. Overall, the obtained *AgNPs*/*γ*-*Al*_2_*O*_3_ materials represent a sustainable and environmentally friendly alternative for photocatalytic wastewater treatment.

The *AgNPs*/*γ*-*Al*_2_*O*_3_ materials obtained represent an environmentally sustainable alternative for the photocatalytic treatment of wastewater containing methyl orange. Although this study provides a preliminary assessment of photocatalytic efficiency at a fixed reaction time point (60 min), future work will examine degradation kinetic profiles and evaluate the stability and reusability of these materials.

## Figures and Tables

**Figure 1:**
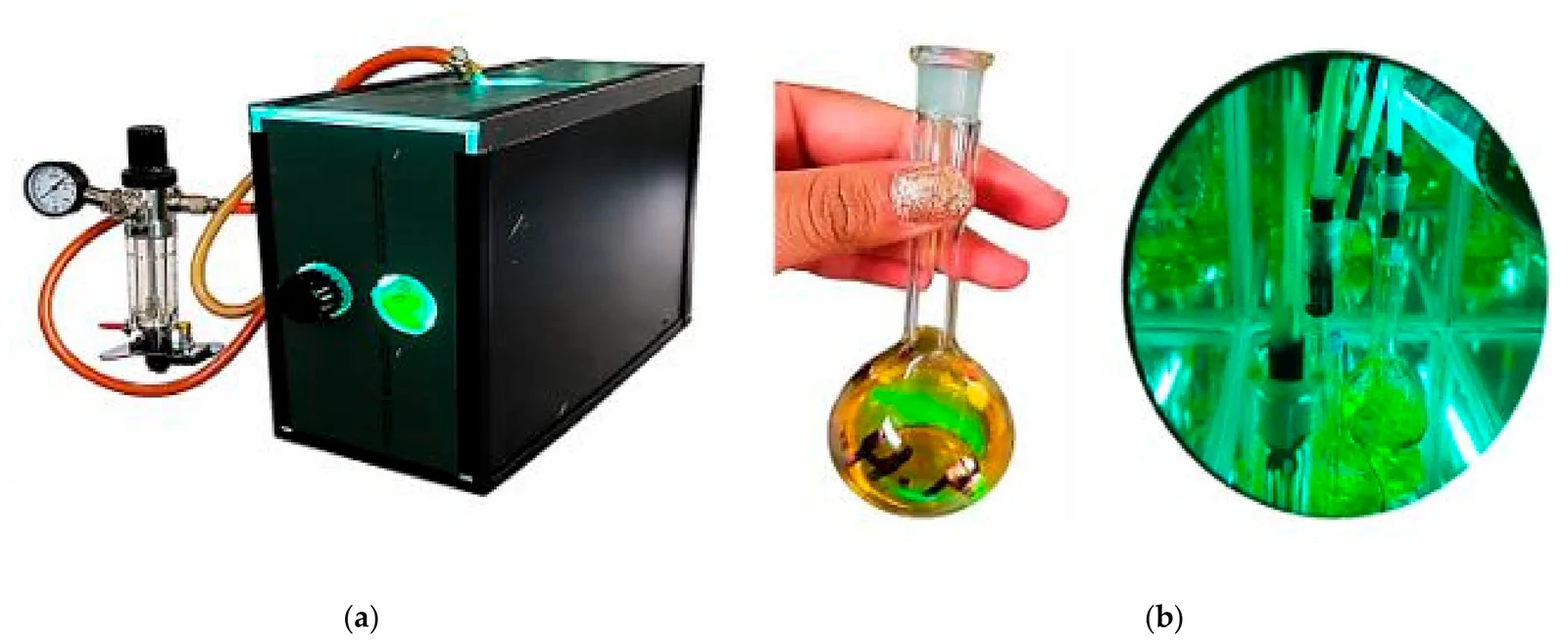
System used for the photocatalytic process. (**a**) Mirror-based photocatalytic reactor employed under UV and white light LED irradiation. (**b**) *AgNPs*/*γ*-*Al*_2_*O*_3_ photocatalyst samples synthesized using RL, PCR, and YRP extracts during MO degradation.

**Figure 2:**
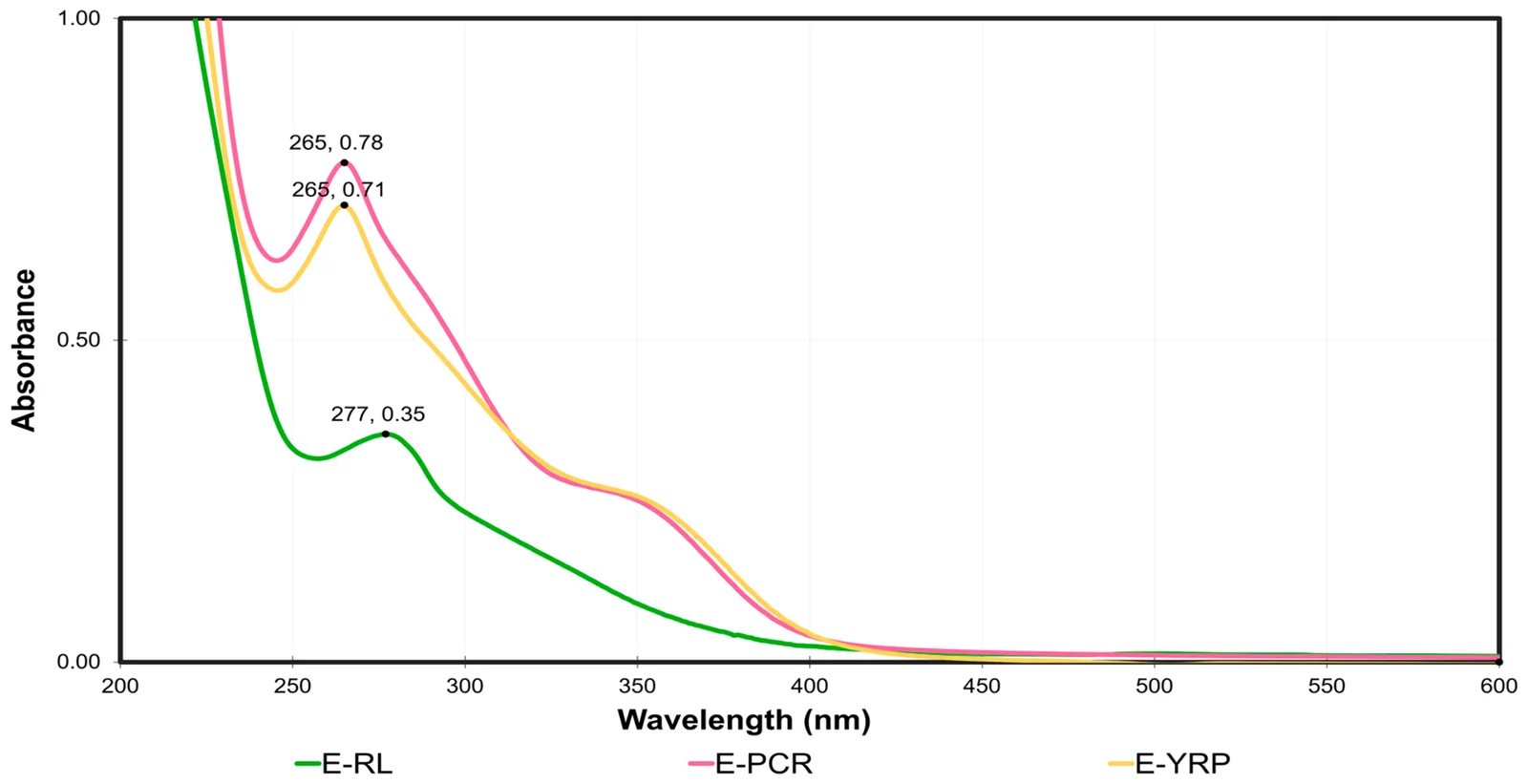
UV-Vis spectra of the 1:100 diluted plant extracts: RL, PCR, and YRP.

**Figure 3:**
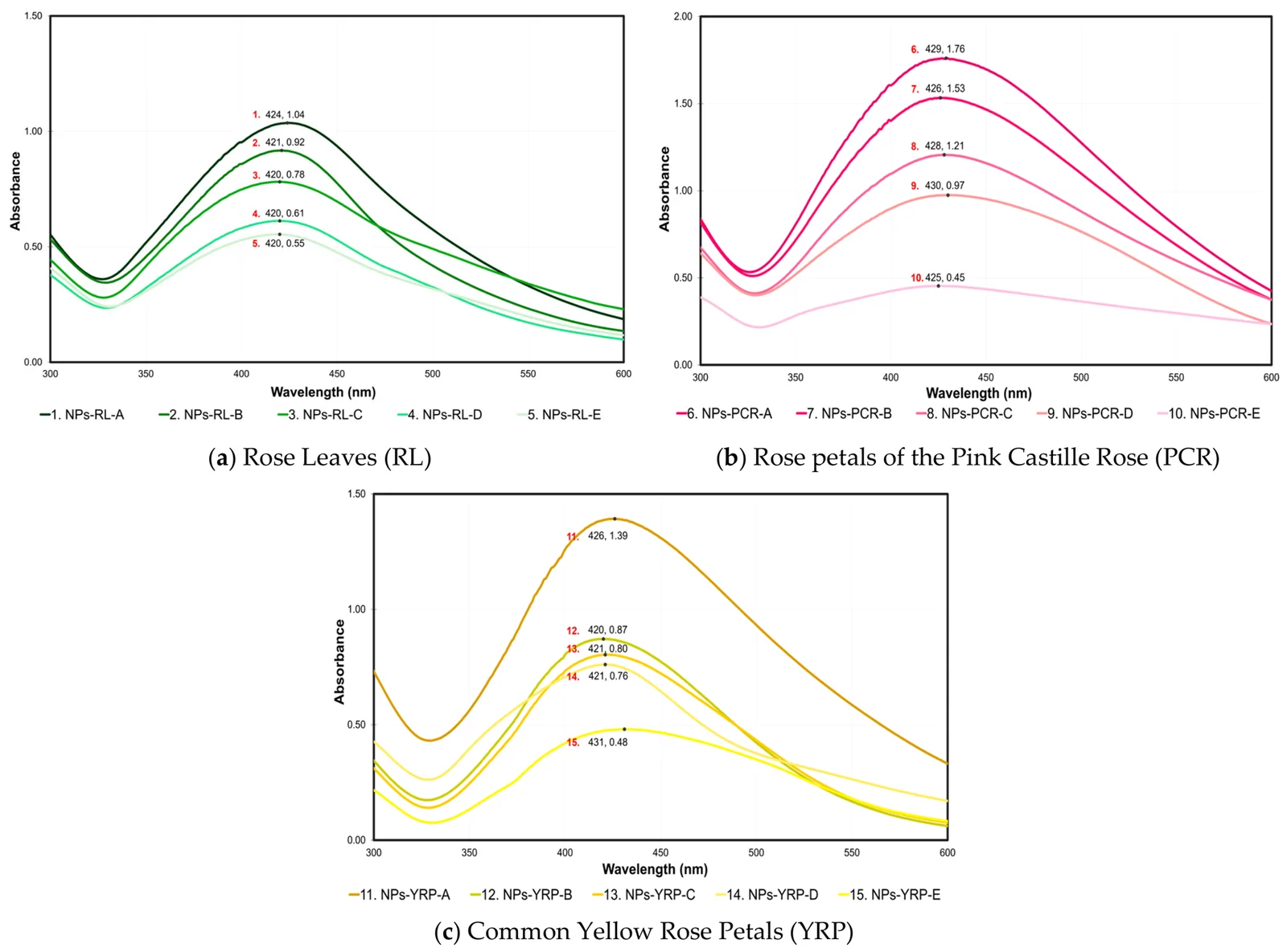
Experimental UV-Vis spectra of AgNPs synthesized using different volumetric ratios of plant extract to AgNO_3_ solution, as specified in Table 1: (**a**) rose leaves (RL), samples 1–5 (NPs-RL-A–E); (**b**) rose petals of the pink Castile rose (PCR), samples 6–10 (NPs-PCR-A–E); and (**c**) common yellow rose petals (YRP), samples 11–15 (NPs-YRP-A–E).

**Figure 4:**
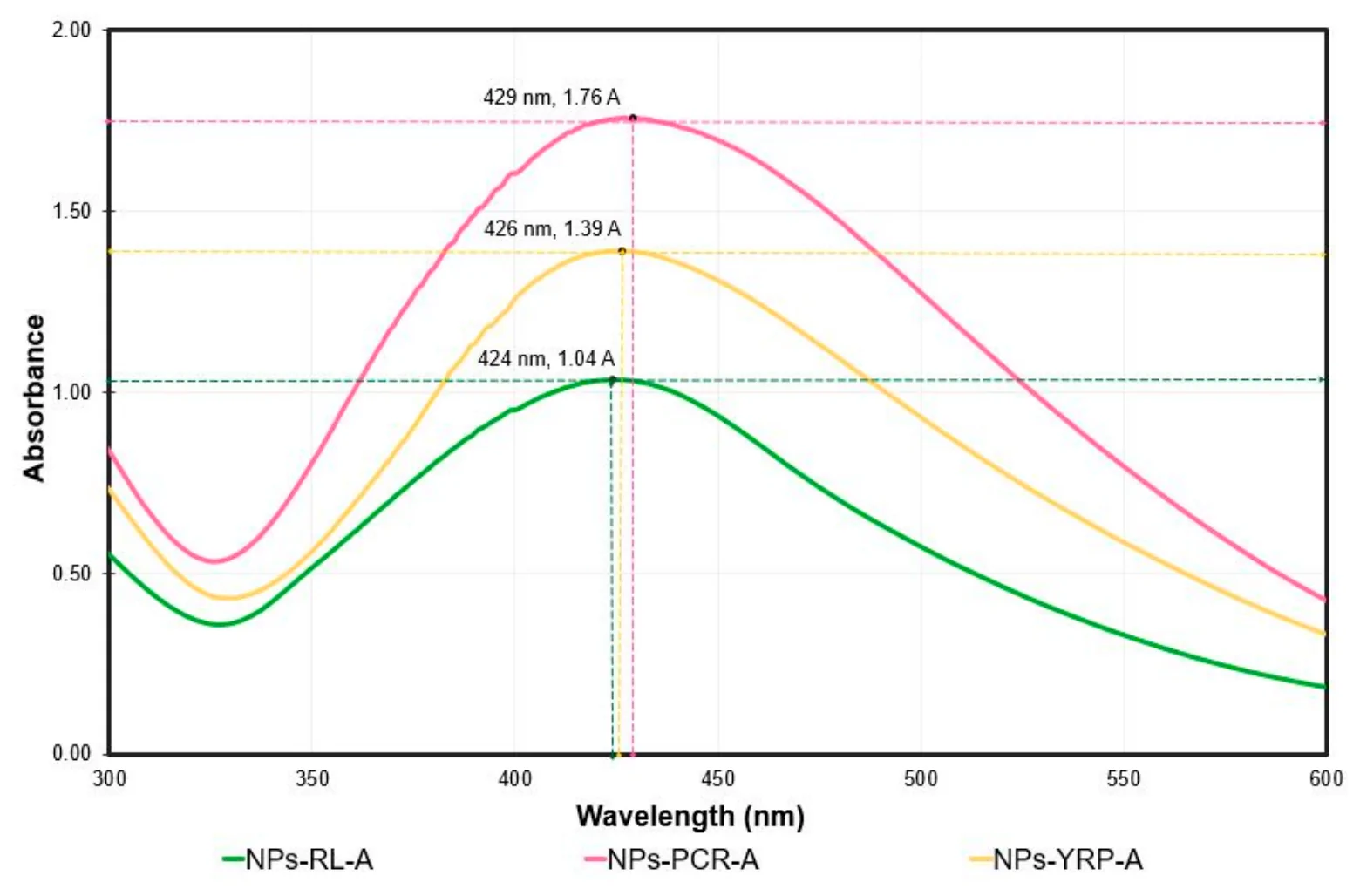
UV-Vis spectra of AgNPs synthesized under the optimized 4:2 extract:AgNO_3_ volumetric ratio using rose leaves (RL; NPs-RL-A, sample 1), rose petals of the pink Castile rose (PCR; NPs-PCR-A, sample 6), and common yellow rose petals (YRP; NPs-YRP-A, sample 11).

**Figure 5:**
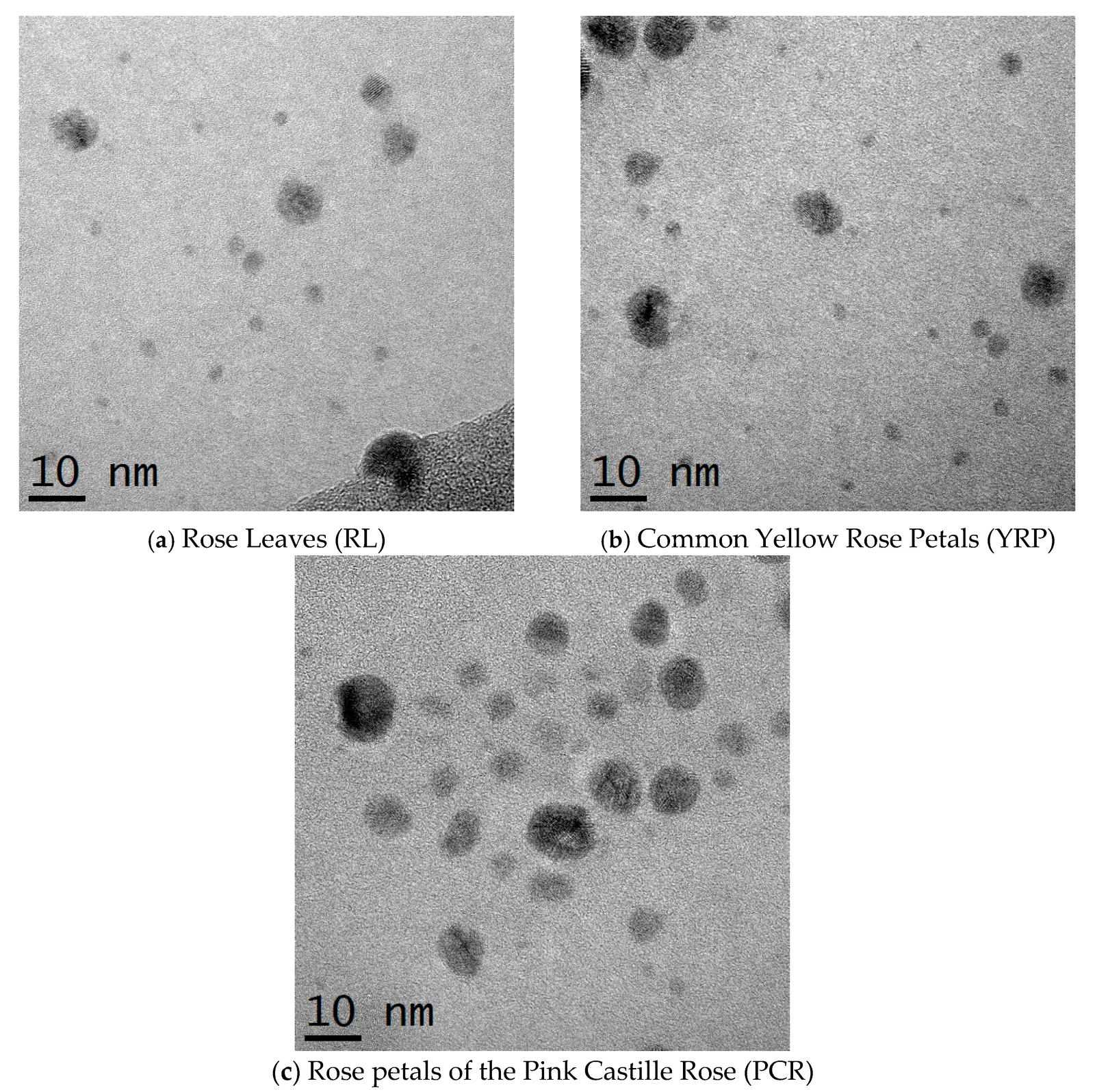
TEM micrographs of the AgNPs synthesized by the green route: (**a**) RL AgNPs, (**b**) YRP AgNPs and (**c**) PCR AgNPs.

**Figure 6:**
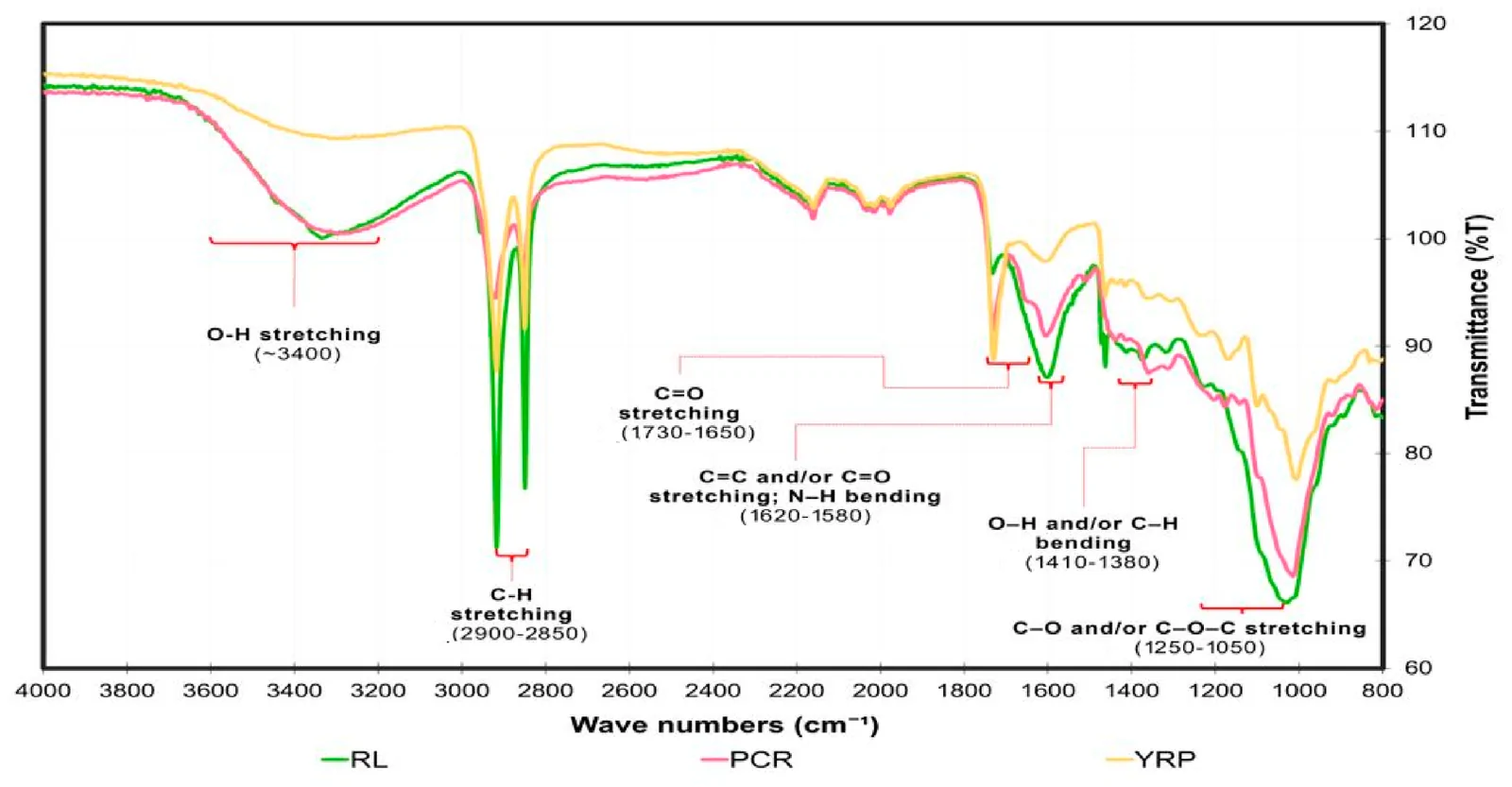
FTIR spectra of the dried plant samples RL, PCR, and YRP in the 4000–800 cm^−1^ region, showing the main absorption bands and their corresponding characteristic functional groups.

**Figure 7:**
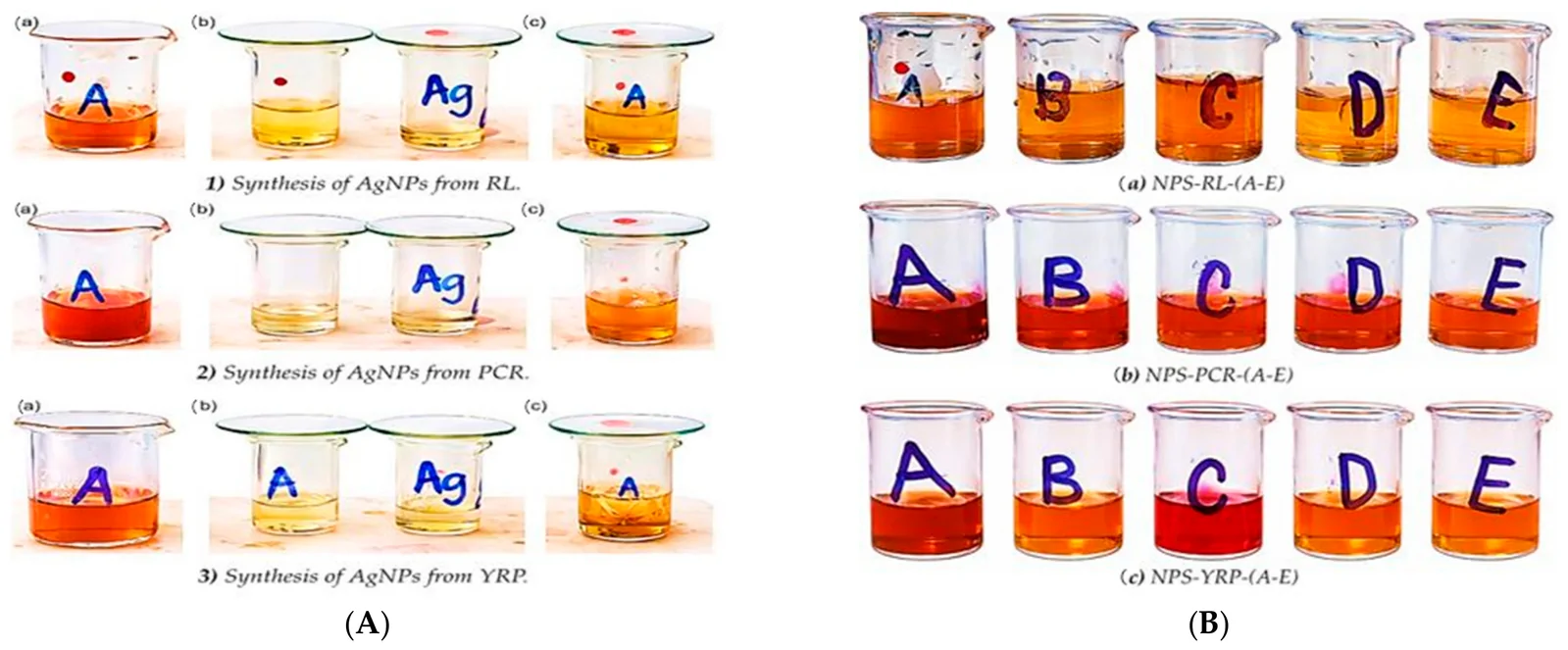
Green synthesis and visual appearance of silver nanoparticles (AgNPs) obtained from RL, PCR, and YRP extracts. (**A**) Synthesis of AgNPs from RL, PCR, and YRP. For each extract: (**a**) stock; (**b**) 1:100 dilution after *AgNO*_3_ addition and heating; (**c**) stirring for AgNP formation. (**B**) AgNP solutions synthesized using the extract: *AgNO*_3_ rations listed in Table 1 for RL, PCR, and YRP.

**Figure 8:**
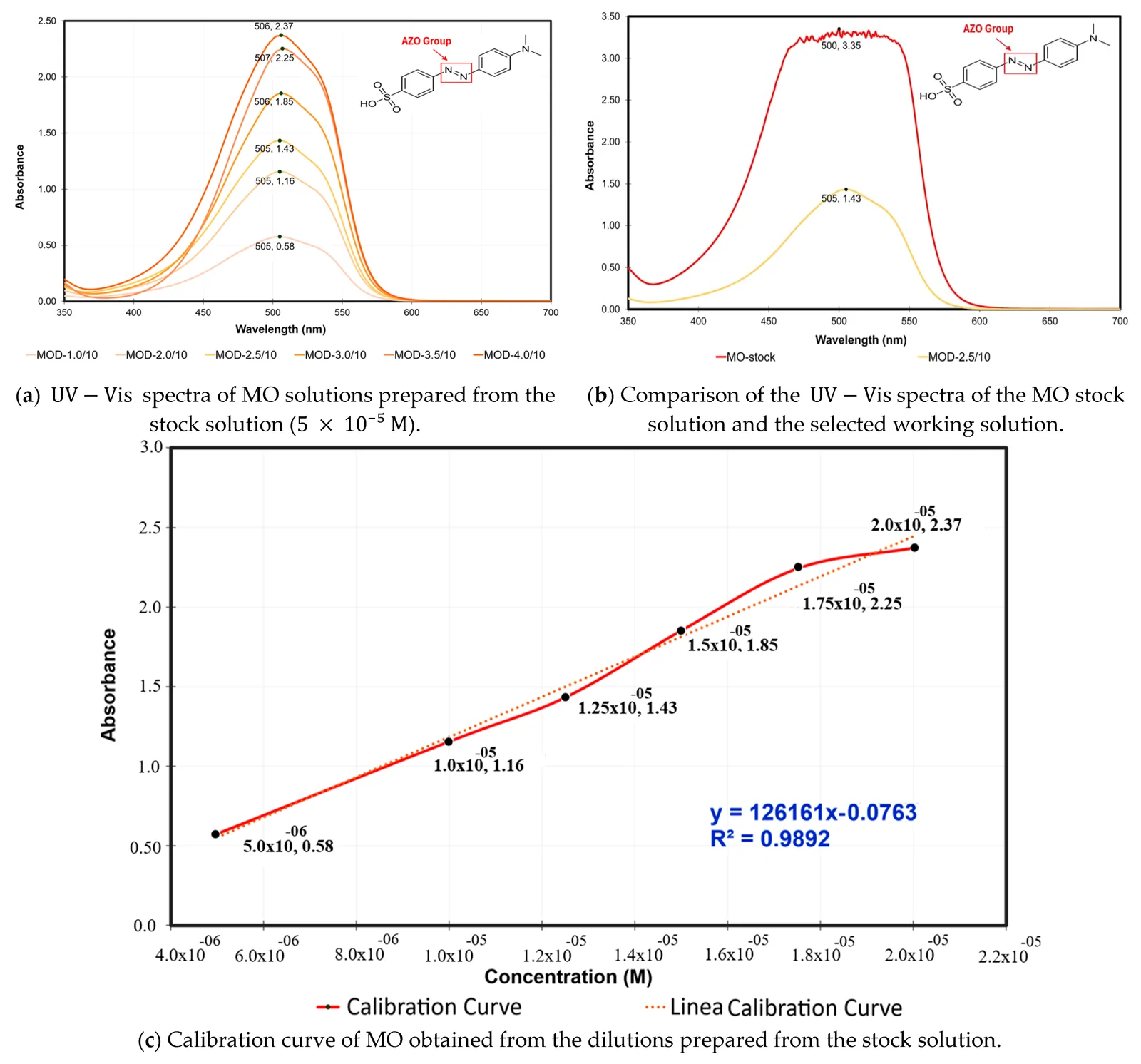
Spectroscopic characterization of the methyl orange (MO) solutions for working concentration selection: (**a**) UV-Vis spectra of dilution showing saturation for MO-stock, (**b**) Comparison highlighting the flat saturated band of MO-stock (*A* > 3.0) versus the peak of the working solution (MOD-2.5/10); (**c**) Calibration curve (R^2^ = 0.9892).

**Figure 9:**
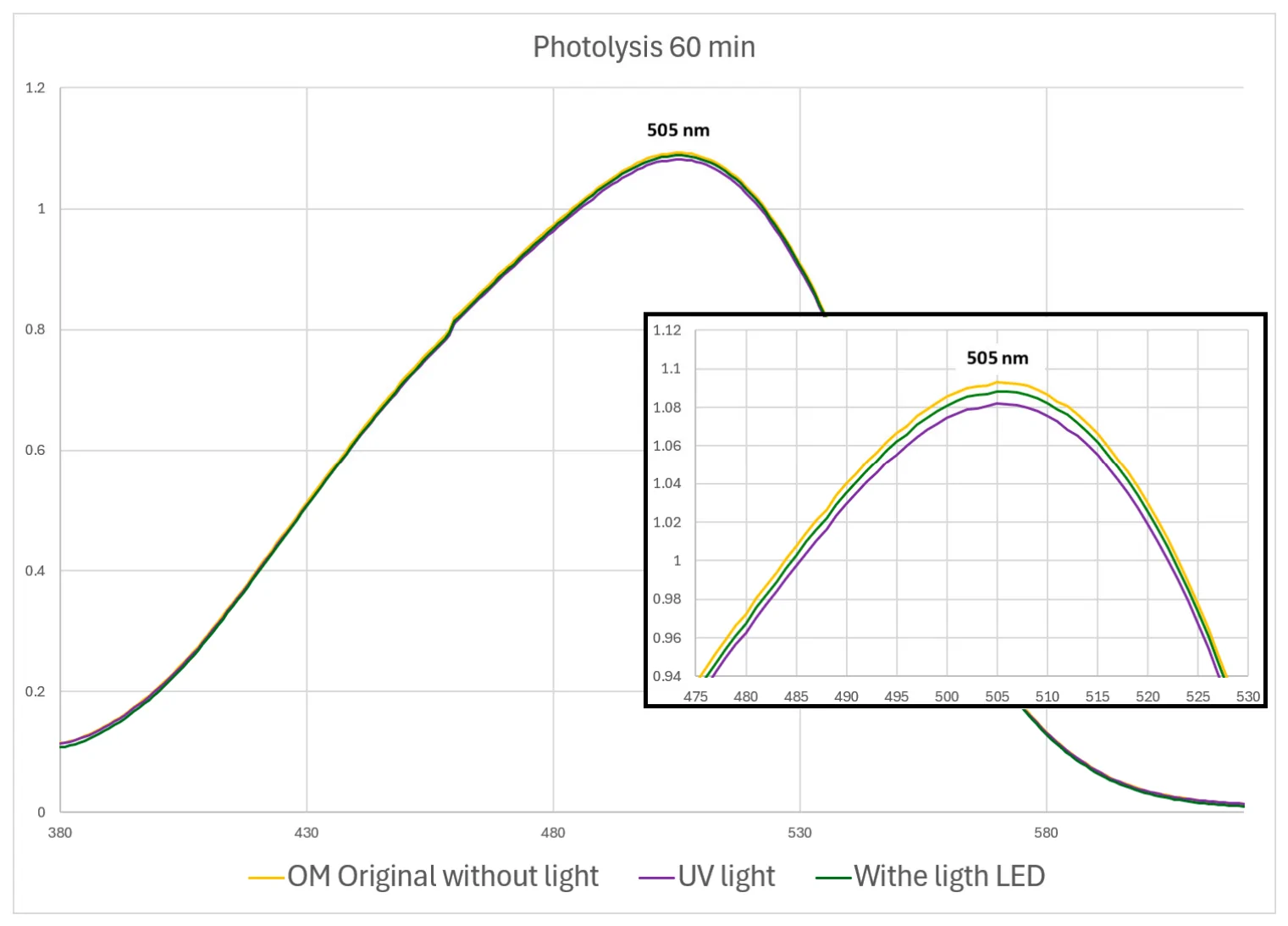
UV-Vis spectrum of OM after 60 min of photolysis.

**Figure 10:**
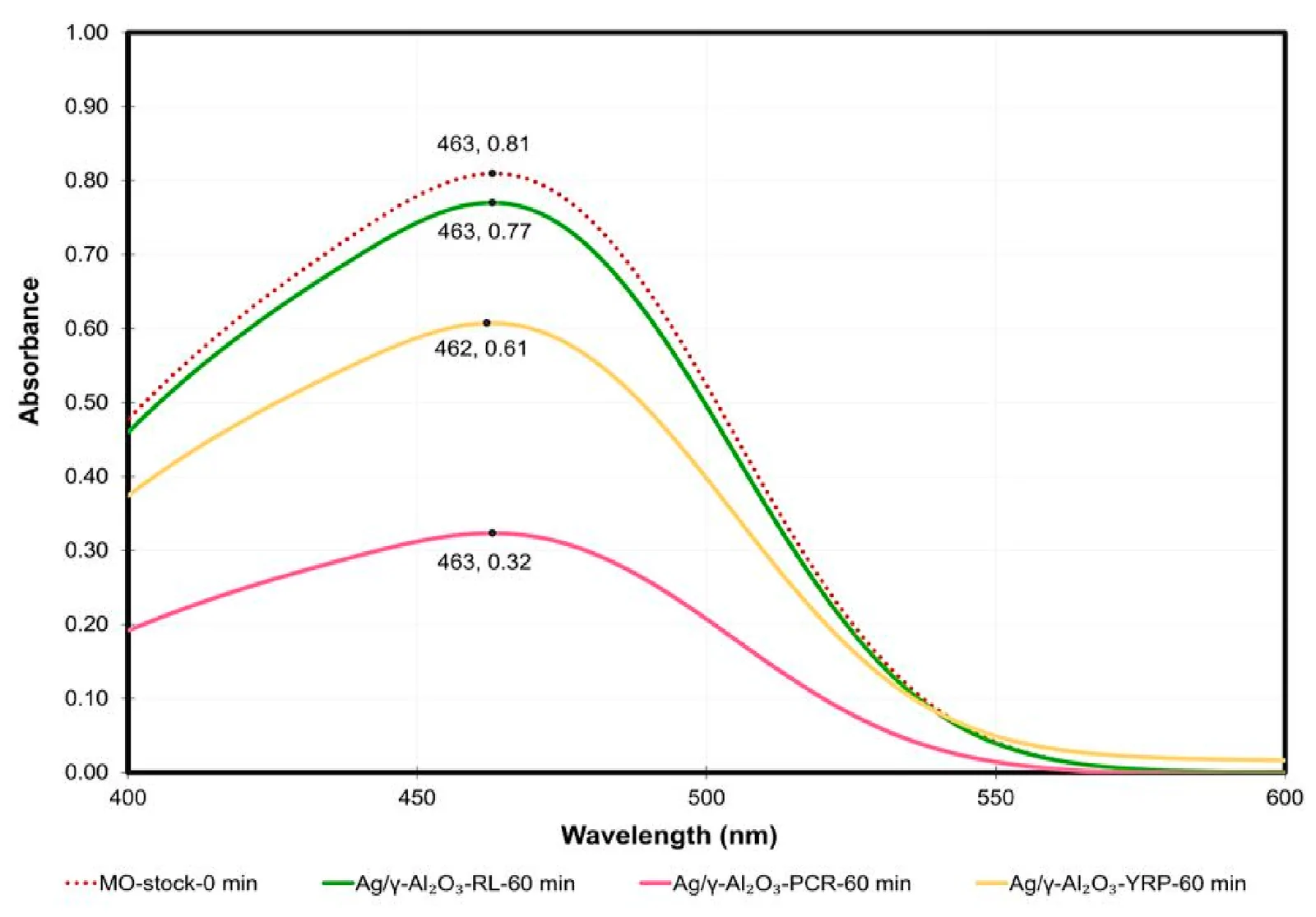
*UV*-*Vis* spectra of methyl orange (MO) before and after 60 min (UV lamp) of photocatalytic treatment with *AgNPs*/*γ*-*Al*_2_*O*_3_ composites obtained from RL, PCR, and YRP extracts.

**Figure 11:**
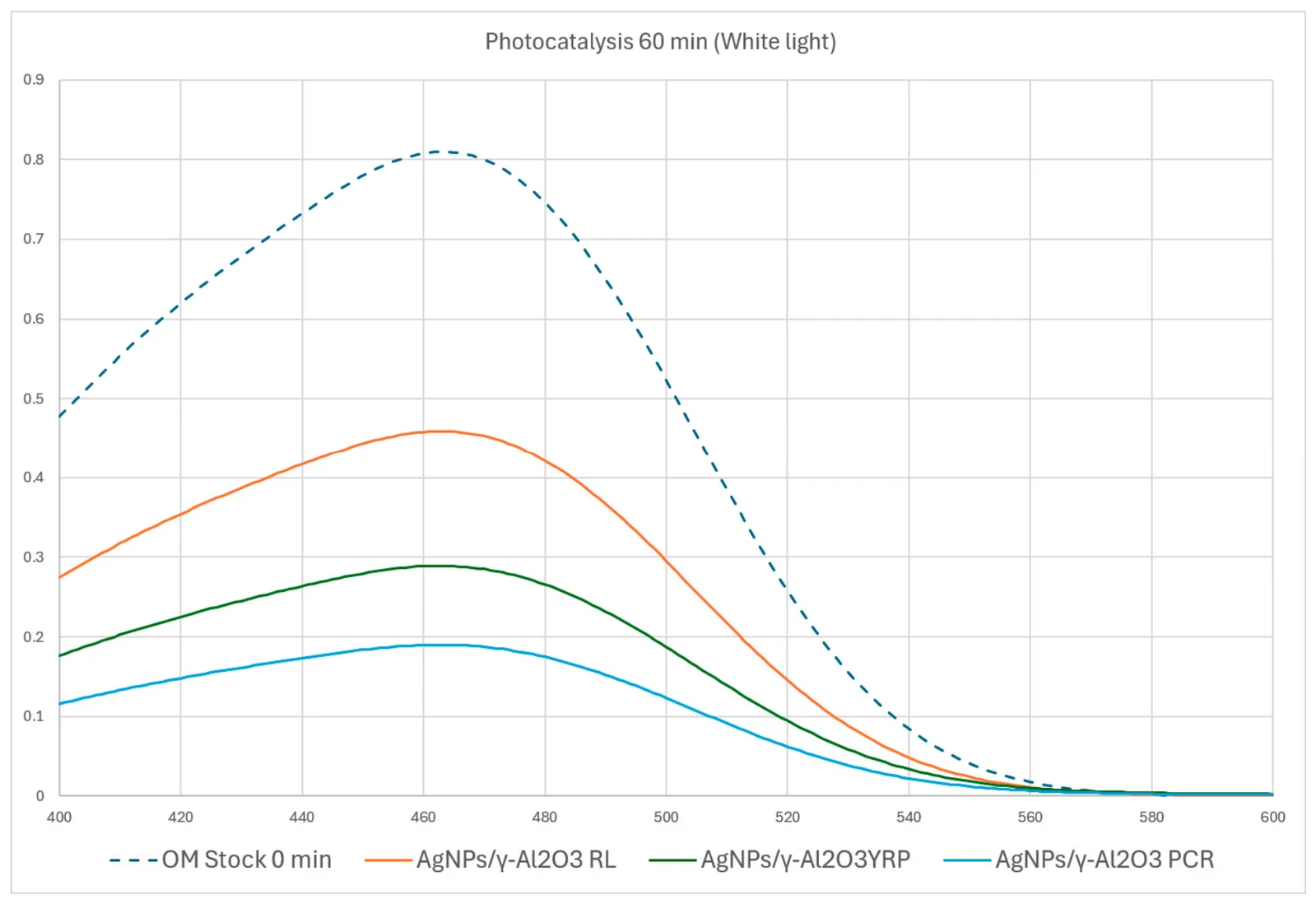
*UV*-*Vis* spectra of methyl orange (MO) before and after 60 min (white LED) of photocatalytic treatment with *AgNPs*/*γ*-*Al*_2_*O*_3_ composites obtained from RL, PCR, and YRP extracts.

**Table 1:** Volumetric ratios of plant extract and AgNO_3_ solution used for the synthesis of AgNPs.

Plant Extract	Sample No.	Sample ID	Extract Volume (mL)	AgNO_3_ Solution Volume (mL)
Rose leaves (RL)	1	NPs-RL-A	4	2
	2	NPs-RL-B	4	3
	3	NPs-RL-C	4	4
	4	NPs-RL-D	3	4
	5	NPs-RL-E	2	4
Rose petals of the pink Castile rose (PCR)	6	NPs-PCR-A	4	2
	7	NPs-PCR-B	4	3
	8	NPs-PCR-C	4	4
	9	NPs-PCR-D	3	4
	10	NPs-PCR-E	2	4
Common yellow rose petals (YRP)	11	NPs-YRP-A	4	2
	12	NPs-YRP-B	4	3
	13	NPs-YRP-C	4	4
	14	NPs-YRP-D	3	4
	15	NPs-YRP-E	2	4

**Table 2:** Dilutions prepared from the methyl orange (MO) stock solution (5 × 10^−5^ M). All solutions were adjusted to a final volume of 10 mL with distilled water. The selected ratio (MOD-2.5/10) is highlighted in bold.

Sample ID	Volume of MO Stock Solution (mL)	Volume of Distilled Water (mL)	Nominal Concentration (M) × 10^−5^	Concentration $\frac{\mathrm{mg}}{L}$
MO-stock	10	0	5.00	16.37
MOD-0.5/10	0.5	9.5	0.25	0.82
MOD-1.0/10	1.0	9.0	0.5	1.64
MOD-2.0/10	2.0	8.0	1.00	3.27
**MOD-2.5/10**	**2.5**	**7.5**	**1.25**	**4.09**
MOD-3.0/10	3.0	7.0	1.50	4.91
MOD-3.5/10	3.5	6.5	1.75	5.73
MOD-4.0/10	4.0	6.0	**2.00**	6.55

**Table 3:** Comparative experimental absorbance values and calculated photocatalytic degradation efficiencies (%) of methyl orange (MO) (*λ_Max_* = 463 nm) under UV and white light LED irradiation after 60 min of treatment.

Sample ID	Experimental Absorbance (A) with UV Light	Experimental Absorbance (A) with White Light LED	Degradation Efficiency (%) with UV Light	Degradation Efficiency (%) with White Light LED
MO-stock (0 min)	0.81	0.81	0	0
DEGRAD-RL (60 min)	0.77	0.46	4.94	43.21
DEGRAD-YRP (60 min)	0.61	0.29	24.69	64.20
DEGRAD-PCR (60 min)	0.32	0.19	60.49	76.54

## Data Availability

The data presented in this study are available on request from the corresponding author.

## Abbreviations

The following abbreviations are used in this manuscript:

*MO*	Metil orange
*AgNPs*	Silver nanoparticles
*RL*	Rose leaves
*PCR*	Rose petals of the pink Castile rose
*YRP*	Common yellow rose petals
TEM	Transmission Electron Microscopy
*LSPR*	Localized surface plasmon resonance
*γ*-*Al*_2_*O*_3_	Gamma-alumina
*AgNPs*/*γ*-*Al*_2_*O*_3_	Photocatalysts
*ROS*	Reactive oxygen species
*CO*_2_	Carbon dioxide
*H*_2_*O*	Water
*AgNO*_3_	Silver nitrate
*UV*-*Vis*	Ultraviolet–visible spectroscopy
*FTIR*	Fourier transform infrared spectroscopy
*λ_max_*	Maximum absorption peaks
*AgNPs*/*γ*-*Al*_2_*O*_3_-*RL*	Photocatalyst based on rose leaves
*AgNPs*/*γ*-*Al*_2_*O*_3_-*PCR*	Photocatalyst based on rose petals of the pink Castile rose
*AgNPs*/*γ*-*Al*_2_*O*_3_-*YPR*	Photocatalyst based on common yellow rose petals
